# Efficacy of expressed breast milk, facilitated tucking, and their combination for pain management during heel stick in preterm neonates: a randomized controlled trial

**DOI:** 10.3389/fped.2025.1622357

**Published:** 2025-09-05

**Authors:** Rupam Das, Vikrant Deshmukh, Arpit Sohane, Pradeep Suryawanshi, Rupeshkumar Deshmukh, Nandini Malshe

**Affiliations:** ^1^ Department of Neonatology, Bharati Vidyapeeth Deemed University Medical College, Pune, India; ^2^ Department of Community Medicine, Bharati Vidyapeeth Deemed University Medical College, Pune, India

**Keywords:** preterm, heel-stick, PIPP score, expressed breast milk, facilitated tucking

## Abstract

**Background:**

Heel-stick procedures for glucose monitoring are common in neonatal intensive care units (NICUs) and can have adverse physiological and developmental effects on preterm neonates. The National Neonatology Forum of India and the American Academy of Pediatrics recommend the routine use of non-pharmacological measures during such procedures. Our study aimed to evaluate the efficacy of expressed breast milk (EBM), facilitated tucking (FT), and their combination in reducing heel stick pain in preterm neonates, as assessed using the Premature Infant Pain Profile (PIPP) score at 1 and 4 min post-procedure.

**Methods:**

In this randomized controlled trial, preterm neonates born between 27 and 36^+6^ weeks of gestation, who met the eligibility criterion, were randomly allocated into three categories: EBM (*n* = 56), FT (*n* = 56), and EBM + FT (*n* = 56), following approval from the Institutional Ethics Committee and parenteral consent at a tertiary-level NICU in Pune. Neonates were videotaped by a senior resident/clinical fellow for 2 min before and up to 4 min after the heel-stick procedure. Infants’ pain was determined using the PIPP score at the time of heel-sticking and at 1 and 4 min post-procedure.

**Results:**

Repeated-measures ANOVA revealed significant reductions in pain scores from baseline (EBM: 8.55 ± 3.19, FT: 8.63 ± 2.83, and EBM + FT: 9.46 ± 2.82; *p* < 0.001) to both 1 min (EBM: 5.68 ± 2.77, FT: 6.39 ± 3.17, and EBM + FT: 6.45 ± 2.87; *p* < 0.001) and 4 min (EBM: 4.05 ± 1.85, FT: 4.66 ± 2.59, and EBM + FT: 4.82 ± 2.39; *p* < 0.001) post-procedure. Bonferroni *post-hoc* analyses confirmed significant within-group reductions in pain across all time points.

**Conclusions:**

EBM and FT, whether alone or in combination, are effective in reducing pain in preterm neonates during heel-stick procedures.

**Clinical Trial Registration:**

https://ctri.nic.in/Clinicaltrials/login.php, identifier [CTRI/2023/09/057787 (Registered on: 18/09/2023)].

## Introduction

During hospital stays, newborns are frequently subjected to invasive and painful procedures, including heel sticks, which might result in adverse physiological, metabolic, or behavioral responses (1). Repeated exposure to pain may also hinder brain development by inducing oxygen desaturation, which leads to the production of free radicals that damage rapidly growing tissues (2, 3). Research has indicated that stress due to neonatal discomfort is related to reduced cognitive and motor functions at corrected ages of 8 and 18 months (4). In addition, such infants may experience increased anxiety, depression, and other behavioral changes at 18 months and even up to 7 years of age (4, 5). These findings necessitate the need for proper interventions to alleviate pain during neonatal intensive care units (NICU) procedures to improve neurodevelopmental outcomes. While numerous pharmacological and non-pharmacological interventions are available for neonatal pain management, the latter are often preferred in preterm infants due to their safety profile and ease of use (6). Expressed breast milk (EBM) represents a promising option, offering advantages such as bioavailability, safety, cost-effectiveness, and the satisfaction a mother feels when she creates an analgesic for her baby (7). There are two possible explanations for the analgesic effect of breast milk: first, its lactose-based sweetness, along with flavor (8) and odor (9), and second, its high tryptophan level (10), which is a melatonin precursor. Beta-endorphin, an endogenous opioid that lessens pain perception, is believed to be released in response to melatonin (11). Similarly, facilitated tucking (FT) involves holding the infant with warm hands to provide tactile and thermal sensory stimulation, thereby helping to lessen pain during invasive procedures (12).

Several pain scoring systems are available for neonates, one of which is the Premature Infant Pain Profile (PIPP; 28–40 weeks), which can be used to assess both procedural and postoperative pain while adjusting for prematurity. Since the PIPP focuses on scoring facial expressions, it is particularly suitable for preterm infants who may have limited motor function and may not be able to fully express pain through crying or movement.

Apaydin Cirik and Efe assessed pain reduction during orogastric tube insertion using the PIPP score and found that the combination of EBM and swaddling produced the maximum analgesic effect (13).

Both the National Neonatology Forum (NNF) of India and the American Academy of Pediatrics (AAP) strongly recommend the routine use of non-pharmacological interventions, such as oral sucrose/glucose solutions, EBM, FT, non-nutritive sucking (NNS), and swaddling, for procedural pain relief in neonates, including during heel pricks (14, 15).

Despite growing emphasis on such strategies, few studies have directly compared the effectiveness of EBM and FT, either individually or in combination, for heel-stick pain relief in preterm neonates. Most available studies have focused on a single intervention or compared these with sucrose, with limited evidence from Indian settings. Moreover, while the NNF claims high-certainty evidence for pain reduction during and up to 30 s after heel pricks, little is known about the sustained effects of these interventions beyond this window. This represents a significant knowledge gap, particularly in resource-limited settings where sucrose may not be readily accessible.

Our study addresses this gap by evaluating the effectiveness of EBM, FT, and their combination in reducing pain during heel-stick procedures using PIPP scores assessed up to 1 and 4 min post-procedure. To our knowledge, no Indian study has compared these three methods. Although a 24% sucrose solution is recognized as an effective technique for pain reduction in preterm newborns before the heel-stick procedure (16–18), it is not readily accessible across Indian markets. In contrast, our proposed alternatives, EBM and FT, are quick, inexpensive, easy to administer, and readily available in every setting and therefore can be used for pain management before the heel-stick procedure.

## Materials and methods

### Study design and participants

A three-arm, parallel-group randomized controlled trial was conducted in a tertiary care NICU of Bharati Vidyapeeth (Deemed University) Medical College, Pune, Maharashtra, India, between October 2023 and May 2024. Preterm neonates aged 27–36^+6^ week gestational age (GA) admitted to the NICU within 48 h of birth were eligible for inclusion. The study was approved by the Institutional Ethics Committee (IEC) and registered with the CTRI **(**CTRI/2023/09/057787). Neonates were excluded if they were on invasive ventilation, hemodynamically unstable, had any major congenital malformations or genetic abnormalities, had encephalopathy, or had received sedatives 24 h prior to the interventions. To assess the intra-class correlation (ICC) of the PIPP score, a pilot study was conducted prior to commencing the main study. The ICC values demonstrated acceptable margins across the EBM, FT, and EBM + FT groups: 0.954 for EBM, 0.985 for FT, and 0.942 for EBM + FT.

### Randomization and sample size

Our sample size was determined using data from a study by Apaydin Cirik and Efe (13), which reported mean PIPP scores of 7.9 and 6.6 in the EBM and EBM + FT groups, respectively, with 31 participants per group. Based on these values, the sample size was calculated to be 56 participants per group, assuming a 95% confidence interval (CI), 80% power, an acceptable error margin of 1.30, a pooled standard deviation (SD) of 2.455, and group-specific SDs of 2.6 for EBM and 2.3 for EBM + FT. Participants were randomly assigned to the EBM, FT, or EBM + FT group using computer-generated numbers. Allocation concealment was ensured using sequentially numbered, opaque, sealed envelopes (SNOSEs). Written informed consent was obtained.

### Interventions

Regardless of the interventions, all infants were positioned supine with rolled towels for support at least 30 min prior to the heel-stick procedure.

**Expressed breast milk group**: A sterile cotton gauze soaked in EBM was placed in the oral cavity of the infant 2 min prior to the procedure and maintained up to 4 min after the heel-stick procedure. The breast milk volume was adjusted as per GA: 0.5 mL for 27–27^+6^ weeks; 1.0 mL for 28–29^+6^ weeks; 1.5 mL for 30–31^+6^ weeks; 2 mL for 32–36^+6^ weeks (19).

**Facilitated tucking group**: Infants were held gently by a qualified nurse or physician with warm hands for 2 min before, during, and up to 4 min after the heel-stick procedure. The infant was positioned in a flexed, midline posture with the limbs adjacent to their body while constraining the head and body.

**EBM** **+** **FT group:** Both interventions, as mentioned above, were applied concurrently.

### Heel-stick procedure and pain assessment

**Heel-stick procedure:** Heel sticks were performed by a neonatal nurse using proper aseptic precautions. An Accusure™ safety lancet (Microgene Diagnostic Systems Private Limited, India) of 28 gauge was used for the procedure. To collect the blood sample, gentle pressure was applied without squeezing the heel after the heel stick.

**Pain assessment:** PIPP was utilized to evaluate procedural pain during heel sticks. Scoring was performed by the principal investigator using video recordings of the infants' facial expressions captured during the heel-stick procedure and at 1 and 4 min post-procedure. Videos were recorded by NICU on-call personnel (senior resident/clinical fellow) who were proficiently trained in capturing the facial expressions of newborns. Concurrently, heart rate and oxygen saturation visible displayed on the bedside pulse oximeter were documented. Due to the nature of the interventions, blinding of the personnel administering the interventions and assessing the PIPP scores was not feasible. Pain indicators were assessed in the following sequence: GA, heart rate, sleep/wake state, oxygen saturation, eye squeeze, brow bulge, and nasolabial furrow. Gestational age was determined using first-trimester dating scans or the last menstrual period; when these were unavailable, the New Ballard Scoring System was employed.

**Physiological parameters:** Heart rate and oxygen saturation were continuously monitored using bedside pulse oximeters. An oxygen saturation probe was affixed to the right upper limb of each infant. Baseline readings were documented before the heel-stick procedure.

## Statistical analysis

Statistical analyses were conducted using SPSS version 29 software. Descriptive statistics such as mean and standard deviation summarized quantitative variables (GA, birth weight, heart rate, SpO_2_, age/time at heel stick, and PIPP score), while frequency and percentages described categorical variables (gender and mode of delivery). Using repeated-measures ANOVA, we analyzed the significant mean change in PIPP scores from baseline to 1 min and up to 4 min post-procedure within each intervention group. Pairwise comparisons were performed using Bonferroni *post-hoc* analysis. ANOVA was used to assess the mean difference in PIPP scores from baseline to 1 min and from baseline to 4 min between the intervention groups. A *P*-value <0.05 was considered statistically significant.

## Results

### Infant characteristics

**Figure 1:**
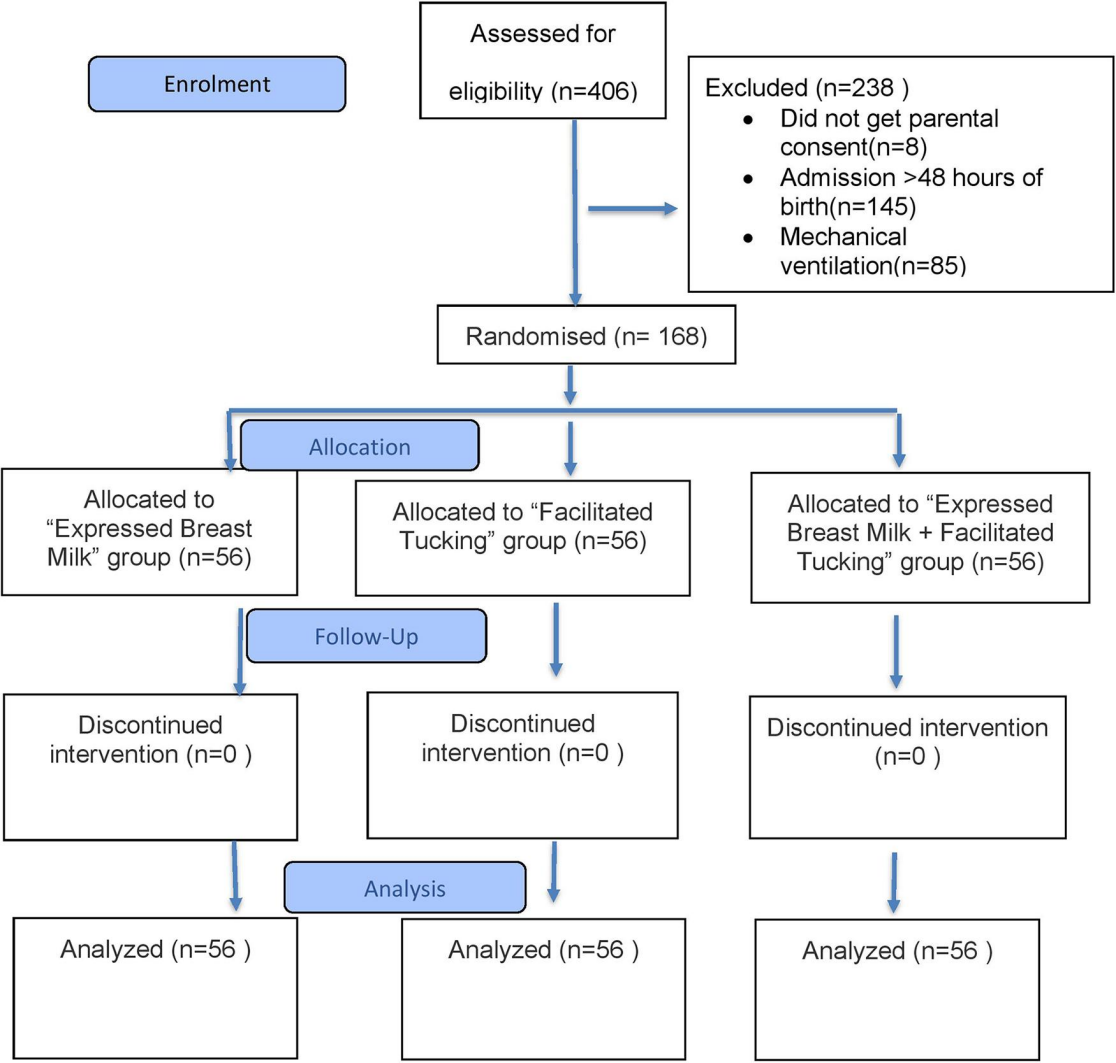
CONSORT 2025 flow diagram.

A total of 406 preterm neonates were assessed for eligibility, of whom 238 were excluded and 168 were enrolled in the study (Figure 1). No participants were lost to follow-up or discontinued from the allocated intervention. The mean gestational ages were 33.57 ± 2.46, 34.26 ± 2.05, and 33.97 ± 2.14 weeks in the EBM, FT, and EBM + FT groups, respectively. The mean birth weights were 1.88 ± 0.54, 1.94 ± 0.58, and 1.96 ± 0.65 kg in the EBM, FT, and EBM + FT groups, respectively. Baseline characteristics of infants were comparable across the three groups, with no statistically significant differences except for baseline heart rate and baseline oxygen saturation (Table 1), which, however, were not clinically significant.

**Table 1:** Baseline characteristics of participants in the groups.

Parameters		EBM (*n* = 56)		FT (*n* = 56)		EBM + FT (*n* = 56)		Chi-square/*F*-value	*P*-value
		Mean	SD	Mean	SD	Mean	SD		
Maternal age		27.57	+5.65	28.38	+4.78	29.14	+4.69	*F*-value = 1.35	0.26
Primigravida, *n* (%)		33 (58.93)		27 (48.21)		25 (44.64)		Chi-square = 2.48	0.29
Multigravida, *n* (%)		23 (41.07)		29 (51.79)		31 (55.36)			
Gestational age		33.57	+2.46	34.26	+2.05	33.87	+2.14	*F*-value = 1.36	0.26
Birth weight		1.88	+0.54	1.94	+0.58	1.96	+0.65	*F*-value = 0.28	0.76
Mode of delivery	LSCS, *n *(%)	41 (73.2)		46 (82.1)		48 (85.7)		Chi-square = 2.94	0.23
	SVD, *n *(%)	15 (26.8)		10 (17.9)		8 (14.3)			
Gender	Female, *n *(%)	19 (33.9)		22 (39.3)		27 (48.2)		Chi-square = 2.42	0.30
	Male, *n *(%)	37 (66.1)		34 (60.7)		29 (51.8)			
Age at time of heel-stick procedures (h)		14.93	+10.12	14.63	+9.39	17.68	+11.19	*F*-value = 1.51	0.23
Baseline heart rate (bpm)		152.14	+11.65	145.29	+14.20	150.39	+15.01	*F*-value = 3.792	0.025
Baseline SPO_2_ (%)		94.48	+2.56	92.89	+2.83	92.89	+3.36	*F*-value = 5.468	0.005

LSCS, lower segment caesarean section; SVD, spontaneous vaginal delivery.

### Differences in pain measurement parameters at the time of heel stick and at 1 and 4 min post-procedure

**Figure 2:**
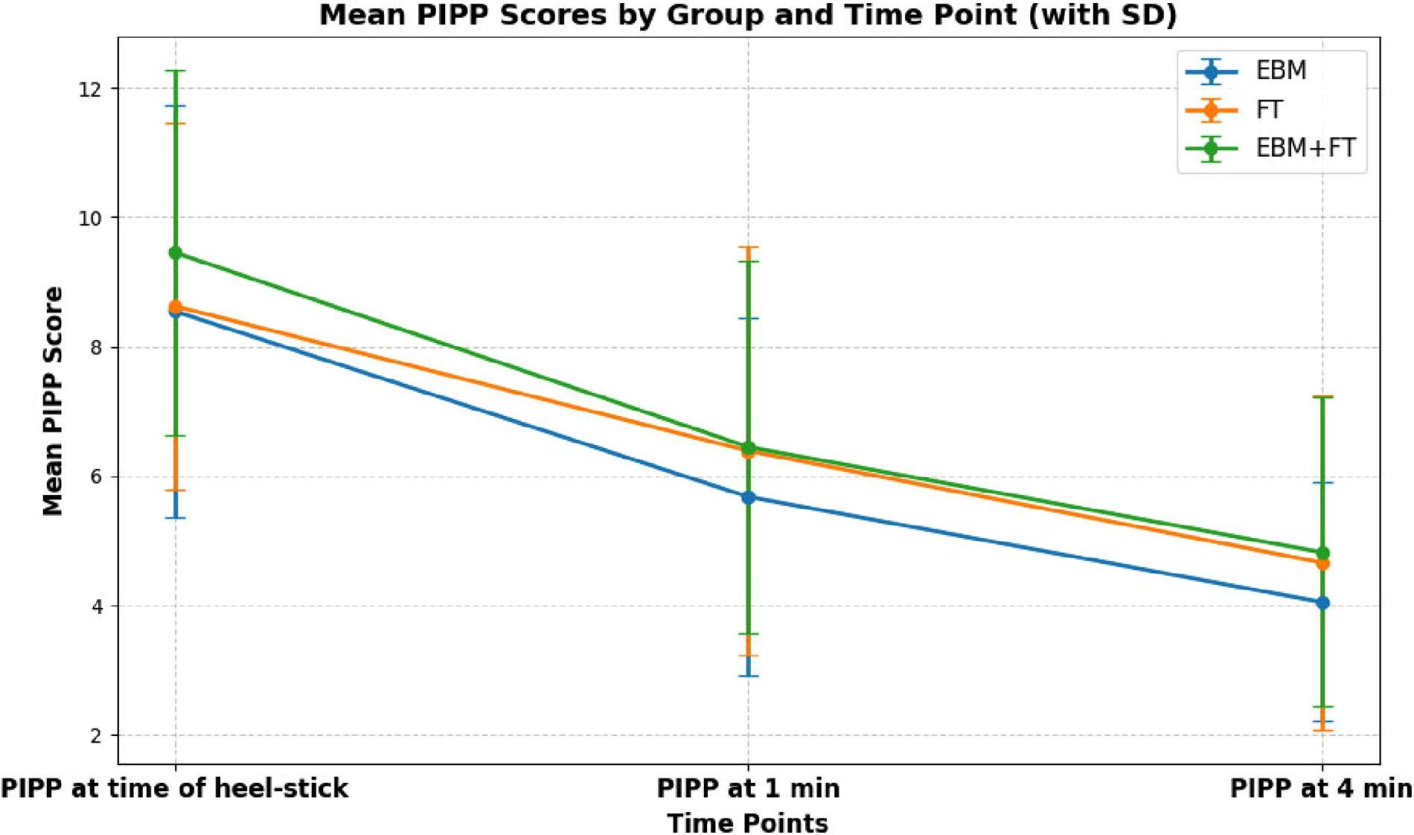
Comparison of preterm infants' PIPP scores in the groups over time.

The efficacy of EBM, FT, and EBM + FT in minimizing pain during heel-stick procedures was examined. Table 2 shows that the mean PIPP score decreased significantly in all three intervention groups from baseline to 1 and 4 min after the heel-stick procedure. This indicates that all three methods are equally effective in reducing pain, as depicted graphically in Figure 2.

**Table 2:** Repeated-measures ANOVA.

Intervention		Mean	SD	*N*	*P*-value
EBM	PIPP at the time of heel-stick procedures	8.55	±3.19	56	<0.001
	PIPP at 1 min	5.68	±2.77	56	
	PIPP at 4 min	4.05	±1.85	56	
FT	PIPP at the time of heel-stick procedures	8.63	±2.83	56	<0.001
	PIPP at 1 min	6.39	±3.17	56	
	PIPP at 4 min	4.66	±2.59	56	
EBM + FT	PIPP at the time of heel-stick procedures	9.46	±2.82	56	<0.001
	PIPP at 1 min	6.45	±2.87	56	
	PIPP at 4 min	4.82	±2.39	56	

Further Bonferroni *post-hoc* pairwise analysis (Table 3) demonstrated that the mean difference in PIPP scores decreased statistically significantly *within each intervention group* across all time points: from baseline (i.e., from time of heel stick) to 1 min, from 1 to 4 min, and from baseline to 4 min*.* Subgroup analyses were not performed because stratified randomization by gestational age was not conducted.

**Table 3:** Bonferroni *post-hoc* pairwise comparisons.

Intervention			Mean difference (*I* − *J*)	SE	*P*-value
EBM	PIPP at the time of heel-stick procedures	PIPP at 1 min	2.875^a^	0.368	<0.001
		PIPP at 4 min	4.500^a^	0.365	<0.001
	PIPP at 1 min	PIPP at 4 min	1.625^a^	0.276	<0.001
FT	PIPP at the time of heel-stick procedures	PIPP at 1 min	3.018^a^	0.357	<0.001
		PIPP at 4 min	4.643^a^	0.446	<0.001
	PIPP at 1 min	PIPP at 4 min	1.625^a^	0.325	<0.001
EBM + FT	PIPP at the time of heel-stick procedures	PIPP at 1 min	2.232^a^	0.257	<0.001
		PIPP at 4 min	3.964^a^	0.345	<0.001
	PIPP at 1 min	PIPP at 4 min	1.732^a^	0.349	<0.001

Based on estimated marginal means.

^a^ The mean difference is significant at the 0.05 level.

No adverse events or complications were reported in any of the groups.

## Discussion

Pain management during procedures such as heel sticks is essential because this may have detrimental physical and developmental effects on preterm newborns in the short and long term (4, 5). The present research examined the effectiveness of EBM and FT, used either alone or in combination, in reducing pain in preterm infants' (as determined by PIPP scores) at various time points after the heel-stick procedure. According to our research, EBM and FT, either used alone or in combination, effectively reduced pain in preterms.

Our results align with those of Peng et al., who reported that combinations of NNS, EBM, and FT significantly reduced pain during heel-stick procedures, as assessed by PIPP scores, compared to routine care (19). While their study evaluated multi-modal interventions, ours was specifically focused on the independent and combined effects of EBM and FT. To the best of our knowledge, this is the first study from India that solely investigated the synergistic analgesic effect of EBM combined with FT for preterm neonates undergoing heel-stick procedures.

Multiple studies have supported the analgesic efficacy of breast milk. Ribeiro et al. reported that breast milk was nearly as effective as sucrose in reducing pain during ophthalmoscopy for retinopathy of prematurity (20). Simomse et al., in their study, found no discernible difference in mean PIPP scores between newborns receiving sucrose (5.5) and breast milk (6.1), with a mean difference of 0.6 (95% CI −1.6 to 2.8; *P* = 0.58) (21). The analgesic effect of EBM was further reinforced in the study by Upadhyay et al., who found that feeding 5 mL of EBM before venepuncture effectively reduced pain in term neonates, as assessed by the Neonatal Facial Coding System (NFCS) scores (22).

However, other studies have suggested that sweet solutions like sucrose or dextrose might be more effective than EBM. Bueno et al. reported poorer effects of EBM compared to 24% sucrose during the heel-stick procedure, as assessed via PIPP scores and crying time (23). Similarly, Sahoo et al. found that 25% dextrose was more effective than EBM in lowering the mean PIPP scores following venipuncture (24).

Facilitated tucking has also been validated as an effective intervention for pain reduction in neonates. Studies by Reyhani et al., Lopez et al., and Ranjbar et al. demonstrated that FT significantly reduces pain in preterm neonates undergoing painful procedures (25–27). The findings of our study are consistent with this evidence and suggest that FT, being non-invasive and easy to apply, represents a robust intervention for pain management in preterm neonates.

Differences across studies may arise from variability in pain assessment tools, choice of comparison groups, type of procedural pain evaluated, and the kind of intervention combinations tested. Also, some studies assessed pain only during or immediately after the procedure, while our study evaluated for an extended time of 4 min.

Our findings are consistent with the broader literature supporting non-pharmacological approaches. A Cochrane review by Stevens et al. reported that 24% sucrose, administered alone or in combination with other interventions such as NNS, significantly reduced procedural pain during heel lancing in preterm neonates, as measured by PIPP scores at both 30 and 60 s post-procedure (17). However, this benefit was not evaluated beyond 1 min, and long-term effects remain unclear. Unlike many of those trials, our study extended the assessment window to 4 min post-intervention, demonstrating sustained analgesic effects of EBM and FT, thus presenting a novel perspective on prolonged benefit, going beyond the current evidence base and the 30 s window cited in the NNF guidelines.

From a practical perspective, EBM and FT are viable and scalable interventions for Indian NICUs, where 24% sucrose is often either unavailable or inconsistently used. Because these methods do n't require specialized materials, extensive training, or pharmacological medications, they are particularly well-suited for routine pain management in resource-constrained settings.

Our results indicate that FT or EBM, whether used alone or in combination, may be able to provide pain relief to preterm newborns who are unable to be breastfed because of severe sickness or who are unable to obtain oral sucrose. Most Indian NICUs do not have easy access to sucrose products, even though oral sucrose, either by itself or in combination with NNS, has been demonstrated to alleviate procedural pain in preterm infants (28, 29) along with their crying/fussy state (30). The results of our study offer a substitute for sucrose in treating pain in preterm neonates.

Our study adds to the growing body of evidence and supports integrating EBM and FT into national neonatal care protocols while also advocating for further multi-center studies and implementation research to scale these interventions widely.

## Strengths and limitations

Our investigation offers several advantages. This randomized controlled study was conducted using a sizable enough sample, employed standardized pain scoring through video recordings, and adhered to strict inclusion/exclusion criteria. To reduce mistakes, a senior resident or clinical fellow with extensive training performed the video recording. To the best of our knowledge, this is the first study from India to evaluate the combined effects of EBM and FT during the heel-stick procedure. These non-pharmacological interventions are quick, easy to learn, and require only a short training session for neonatal nurses. Thus, these interventions can be applied even at peripheral health centers catering to newborn care. Despite its strength, the study was not without limitations. It was conducted at a single center. Gestational age-wise stratification was not performed, hindering subgroup analyses. Blinding the principal investigator to the allotted interventions while assessing the PIPP score was not feasible. Also, PIPP scores were not documented before the start of the procedure, making it rather difficult to discern whether the changes were because of pain management or because of decreased stress levels due to intervention. Furthermore, we simply measured pain scores as an outcome. Future research should include other outcome factors, such as oxygen needs and sleep and salivary cortisol levels.

## Conclusion

For preterm newborns experiencing heel-sticking pain, EBM and FT, whether used separately or in combination, demonstrate a favorable analgesic impact. As a result, they can be suggested as a means of managing pain during the heel-stick procedures. In addition, these interventions are low-cost and do not require policy development implications, which increases the likelihood of their use in clinical practice.

## Data Availability

The raw data supporting the conclusions of this article will be made available by the authors, without undue reservation.
